# Pre-aggregation of scalp progenitor dermal and epidermal stem cells activates the WNT pathway and promotes hair follicle formation in in vitro and in vivo systems

**DOI:** 10.1186/s13287-019-1504-6

**Published:** 2019-12-19

**Authors:** Yiqun Su, Jie Wen, Junrong Zhu, Zhiwei Xie, Chang Liu, Chuan Ma, Qun Zhang, Xin Xu, Xunwei Wu

**Affiliations:** 1 Department of Tissue Engineering and Regeneration, School and Hospital of Stomatology, Shandong University, Jinan, China; 2 Shandong Provincial Key Laboratory of Oral Tissue Regeneration & Shandong Engineering Laboratory for Dental Materials and Oral Tissue Regeneration, Jinan, China; 3 Department of Implantology, School and Hospital of Stomatology, Shandong University, Jinan, China; 4 Women and Children’s Hospital of Hubei Province, Wuhan, Hubei China; 5 Department of Stomatology, Shengli Oilfield Central Hospital, Dongying, Shandong China; 6 School of Stomatology, Shandong University, 44-1 Wenhua West Road, Jinan, 250014 Shandong China

## Abstract

**Background:**

Billions of dollars are invested annually by pharmaceutical companies in search of new options for treating hair loss conditions; nevertheless, the challenge remains. One major limitation to hair follicle research is the lack of effective and efficient drug screening systems using human cells. Organoids, three-dimensional in vitro structures derived from stem cells, provide new opportunities for studying organ development, tissue regeneration, and disease pathogenesis. The present study focuses on the formation of human hair follicle organoids.

**Methods:**

Scalp-derived dermal progenitor cells mixed with foreskin-derived epidermal stem cells at a 2:1 ratio aggregated in suspension to form hair follicle-like organoids, which were confirmed by immunostaining of hair follicle markers and by molecular dye labeling assays to analyze dermal and epidermal cell organization in those organoids. The hair-forming potential of organoids was examined using an in vivo transplantation assay.

**Results:**

Pre-aggregation of dermal and epidermal cells enhanced hair follicle formation in vivo*.* In vitro pre-aggregation initiated the interactions of epidermal and dermal progenitor cells resulting in activation of the WNT pathway and the formation of pear-shape structures, named type I aggregates. Cell-tracing analysis showed that the dermal and epidermal cells self-assembled into distinct epidermal and dermal compartments. Histologically, the type I aggregates expressed early hair follicle markers, suggesting the hair peg-like phase of hair follicle morphogenesis. The addition of recombinant WNT3a protein to the medium enhanced the formation of these aggregates, and the Wnt effect could be blocked by the WNT inhibitor, IWP2.

**Conclusions:**

In summary, our system supports the rapid formation of a large number of hair follicle organoids (type I aggregates). This system provides a platform for studying epithelial-mesenchymal interactions, for assessing inductive hair stem cells and for screening compounds that support hair follicle regeneration.

## Background

Stem cells play crucial roles in repairing damaged tissues or organs after injury, because they have the capacity to self-organize and self-assemble into complex and functional tissues and organs [1, 2]. By utilization of this ability, organoids have recently been developed in in vitro cultures under optimal conditions, providing new opportunities to study human development and diseases and tissue regeneration [3–5]. Organoids, three-dimensional structures derived from stem cells that have characteristics similar to actual organs, can be used to understand a wide scope of different research areas [6–8]. Although multiple epithelial organoids, such as mammary glands, salivary glands, and colon and liver ducts, have been successfully generated recently, there are still challenges to generating some other types of organoids such as teeth and hair follicles in vitro [9–11]. Hair follicles are dynamic structures that have a wide range of functions including sensory activity, skin moisture retention, thermoregulation, and esthetic appearance [12]. Recently, Weber et al. successfully regenerated hair peg-like structures from fresh fetal scalp-derived dermal progenitor cells combined with cultured neonatal foreskins using a droplet method in vitro [13]. That was the first report of an in vitro generation of human hair follicle organoids; however, the dermal cells had not been expanded in culture and the droplet method is not practical to generate large-scale organoids for research and clinical applications such as screening assays.

It has been a long-term challenge to reconstitute human hair follicles with culture-expanded cells, but recently several laboratories, including ours, have made progress [14–19]. However, the regeneration efficiency of human hair follicles is still low, especially for cells derived from adult tissues [17]. Therefore, it remains important to develop new technologies and methods to improve the regeneration potential of culture cells both in vitro and in vivo. It has been shown that aggregations of dermal papilla cells (sphere cultures) can maintain their trichogenic potential, and the transplantation of dermal papilla spheres together with epidermal cells enhances the efficiency of hair formation in vivo [20, 21]. It has been well established that the initiation of hair follicle morphogenesis requires a crosstalk between epidermal and mesenchymal cells [12, 22]. During morphogenesis, the signal exchange between epidermal cells and underlying dermal cells initiates epidermal cell aggregation to form the epithelial placode, and sequentially the dermal condensate [23, 24]. Further signals sent between dermal condensates and the overlaying epidermal placodes regulate the behavior of both cell populations and ultimately orchestrate the formation of hair follicles and dermal papillae [22, 24, 25]. Among these signals, which include WNT, SHH, NOTCH, BMP, and other signaling pathways, it has been shown that the Wnt signaling pathway is the master regulator [22, 24–28]. Activation of WNT/β-catenin signaling has been observed both in epithelial and in dermal cells. When WNT/β-catenin signaling is turned off, either by the deletion of β-catenin, by the transgenic expression of a truncated form of LEF1, a downstream target of WNT, or by overexpression of the WNT inhibitor DKK1, the formation of hair follicles is blocked [29–31]. Although the crosstalk between epidermal and dermal cells has been recognized to be essential for hair follicle development, no studies have tested whether pre-aggregating epidermal and dermal cells in vitro can enhance hair formation in vivo. Therefore, the present study was aimed to investigate whether transplantation of pre-aggregating epidermal and dermal cells can enhance hair formation in vivo, to determine whether those cells can aggregate to form hair structures in vitro, and to study the role of the WNT signaling pathway during the aggregation.

## Materials and methods

### Cell preparation

Human fetal dermal (FDer) (derived from EGA (estimated gestational age) 15–18 weeks old scalp tissues) and adult dermal (ADer) (derived from 20 to 60 years old scalp tissues) progenitor cells were derived from frozen aliquots that had been used in our previous study [18], and have been shown to have multiple differentiation potentials [18, 32]. Epidermal progenitor (Epi) cells were isolated from a pool of three newborn foreskin tissues (age 0) and were prepared as previously described [33, 34]. Epi cells at passage 3 were able to form holoclones, a defining characteristic of epidermal stem cells (Additional file 1: Figure S1). Human tissues, including fetal scalp tissue, adult scalp tissue, and foreskin tissue, were collected from discarded hospital specimens without any personal identity information, following methods approved by the Medical Ethical Committee of the School of Stomatology, Shandong University (NO. 2015120401, Date: 12-05-2015). Mouse epidermal progenitor cells were obtained from neonatal C57BL/6 mice following a previously published protocol [35, 36]. Human Epi cells were cultured in K-SFM (Cat. 17,005–042, Thermo Fisher Scientific). Human dermal progenitor cells were cultured in DMEM/F12 (3:1) containing 0.1% penicillin/streptomycin, 40 μg/ml fungizone, 40 ng/ml FGF2, 20 ng/ ml EGF, and 2% B27 supplemented with 5% fetal bovine serum (FBS). Cultured human cells at passage 3 and freshly isolated mouse neonatal epidermal (MNE) cells were used for the following experiments.

### Aggregation assay (suspension culture)

To produce dermal-dermal (dermal cells alone), epidermal-epidermal (epidermal cells alone), or dermal-epidermal (a 1:1 mixture of dermal and epidermal cells) aggregates, dissociated skin progenitor cells were suspended in DMEM/F12 (3:1) culture medium containing 0.1% penicillin/streptomycin, 5 ng/ml FGF2, 5 ng/ml EGF, 1% B27, and 1 mg/ml BPE (Bovine Pituitary Extract) supplemented with 2% FBS. They were then plated into ultra-low attachment Costar®6-well plates (Cat.3471, Corning) at a density of 2 × 10^5^ cells in 2 ml medium per well and were cultured at 37 °C in a tissue culture incubator with 5% CO_2_ to form aggregates. The growth medium was changed every other day. A flowchart of the aggregation assay is shown in Additional file 1: Figure S2. The aggregates were harvested at the desired time points for in vivo or in vitro assays.

For the in vivo assay to test the hair regeneration potential of aggregates, cells under three conditions were prepared according to Table 1. Each condition consisted of three groups as shown in Table 1. A total of 4 × 10^6^ cells from each group were transplanted for each graft.

**Table 1:**
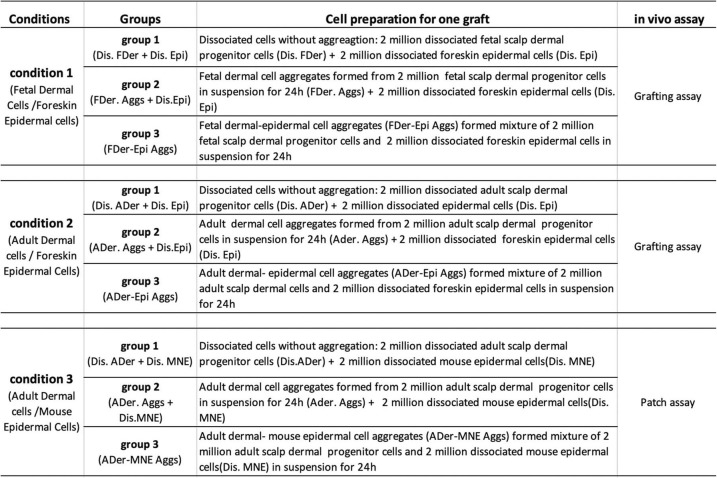
Cell preparation for in vivo assay

For the in vitro assay, a total of 2 × 10^5^ cells (either dermal cells alone, epidermal cells alone, or a mixture of dermal and epidermal cells) were plated in wells of ultra-low adherent six-well plates to form dermal, epidermal, or dermal-epidermal aggregates. To track the localization of epidermal cells in the aggregates, epidermal stem cells were labeled using either Qtracker™ 525 (green, Cat. Q25041MP, Thermo Fisher Scientific) or Qtracker™ 625 (red, Cat. A101098, Thermo Fisher Scientific), and subsequently, scalp dermal progenitor cells were added. The aggregates were harvested for analysis at the desired time points as indicated in Figs. 2, 3, 4, and 5.

### Hair reconstitution assay in vivo

Eight-week-old female *nude/nude* mice (Crl:NU-Foxn1 nu) (Charles River) were used for the in vivo hair reconstitution assays (four mice were used for each group). Animal experiments were performed in accordance with protocols approved by the IACUC of School of Stomatology, Shandong University. The two following different approaches were carried out for the in vivo assays.

1. Grafting assays

To test the hair regeneration ability of human dermal-epidermal aggregations, we performed grafting assays as previously reported [17, 19, 37]. Cells were prepared according to conditions 1 and 2 as shown in Table 1. Cells from each group were collected and resuspended in 100 μL F12 medium and transferred onto PET membranes (Becton Dickinson; Cat. No. 353091). The membranes with cell masses were incubated at 37 °C for 1.5 h in a tissue culture incubator and then grafted onto the dorsal skin of nude/nude mice. Graft area excisions at 3 months after transplantation were quantified for the formation of hair follicles and were processed for histological analysis.

(2) Human-mouse hybrid patch assays

To test the regeneration potential of adult scalp dermal progenitor cells, human-mouse hybrid patch assays were performed according to previous studies [18, 36, 38]. Cells were prepared according to condition 3 in Table 1. Cells from each group were collected and resuspended in 75 μl F12 medium and were subcutaneously injected into the dorsal skin of nude/nude mice; each mouse received four subdermal injections of cells. The “patches” formed were harvested 4 weeks after injection and numbers of hair follicles formed were determined using a dissecting microscope.

### Histological analysis and immunofluorescence (IF) staining

Histological and IF analyses followed standard protocols [17, 37, 39]. Briefly, aggregates were collected and washed with phosphate buffered saline (PBS) containing 0.5% bovine serum albumin (BSA). The reconstituted skin and aggregates were fixed in 4% paraformaldehyde (PFA, Cat. P6148, Sigma) for 30 min at room temperature. The fixed specimens were washed in 0.5% BSA in PBS and were then soaked in 7.5% sucrose in PBS for 3 h at room temperature, and then transferred to 15% sucrose in PBS at 4 °C overnight. They were then embedded in optical cutting temperature (O.C.T) compound (Cat. 23-730-57, Fisher Scientific), frozen at − 80 °C, and then sectioned at 6 μm for histological analysis (hematoxylin and eosin (H&E) stain) and IF staining.

The following primary and secondary antibodies were used for IF analysis: rat anti-FITC conjugated (CD49) α6-integrin (Cat. 1011, Stem Cell); monoclonal mouse anti-human keratin 14/15/16/19 (pan-cytokeratin) (Cat. 550,951, BD); monoclonal mouse anti-human vimentin (5G3F10)(Cat. 3390, Cell Signaling); monoclonal rabbit anti-cytokeratin 14 (K14) (Cat. Ab119695, Abcam); monoclonal mouse anti-cytokeratin 15 (K15) (Cat. ab80522, Abcam); monoclonal rabbit anti-cytokeratin 17 (K17) (Cat. ab51056, Abcam); monoclonal rabbit anti-cytokeratin 6 (K6) (Cat. ab93279, Abcam); polyclonal rabbit anti-versican (Cat. Sc-47,769, Santa Cruz); and polyclonal rabbit anti-Ki67 (Cat. ab15580, Abcam). The following secondary antibodies (all from Life Technologies) were used: Alexa fluor-488 donkey anti-mouse IgG (Cat. A21202); Alexa fluor-594 donkey anti-mouse IgG (Cat. A21203); Alexa fluor-488 donkey anti-rabbit IgG (Cat. A21206); and Alexa fluor-594 donkey anti-rabbit IgG (Cat. A21207). The stained slides were mounted with mounting medium with DAPI (Cat. H-1200, Vector Laboratories).

### Real-time qPCR analysis

Real-time quantitative PCR analysis followed the standard protocol [40]. Briefly, total RNA was extracted from cells or cell aggregates with a QIAGEN RNeasy Plus Mini Kit (QIAGEN, Hilden, Germany) according to the manufacturer’s instructions. Total RNA (1 μg) was reverse transcribed using a Takara PrimeScriptTM RT reagent Kit (Takara Bio Inc.). PCR reactions were performed with Takara SYBR Premix Ex TaqTM II (Takara Bio Inc.) with LightCyclerR 480 II (Roche Diagnostics Ltd.). cDNA (100 ng) with 250 nm specific gene primers in a total of 20 μl qRT-PCR reaction volume was used for amplification. The thermal cycling conditions for PCR reactions included an initial denaturation at 95 °C for 30 s, 40 cycles at 95 °C for 5 s, 55 °C for 30 s, and 72 °C for 15 s. Data were acquired and analyzed using a LightCycler 480 system. PCR primers were synthesized by BioSune (Shanghai, China) and the oligo sequences are listed in Additional file 1: Table S1; 36B4, a housekeeping gene was used as an internal control.

### Statistical analysis

All experiments were repeated three times (*n* = 3) with scalp dermal cells derived from three different donors. Data are expressed as means ± standard deviation (mean ± SD); *P* values from the statistical analyses are indicated in the text and figures. Student’s *t* test was used to analyze differences between two experimental groups.

## Results

### Pre-aggregation of dermal and epidermal cells enhances hair follicle formation in vivo

**Fig. 1:**
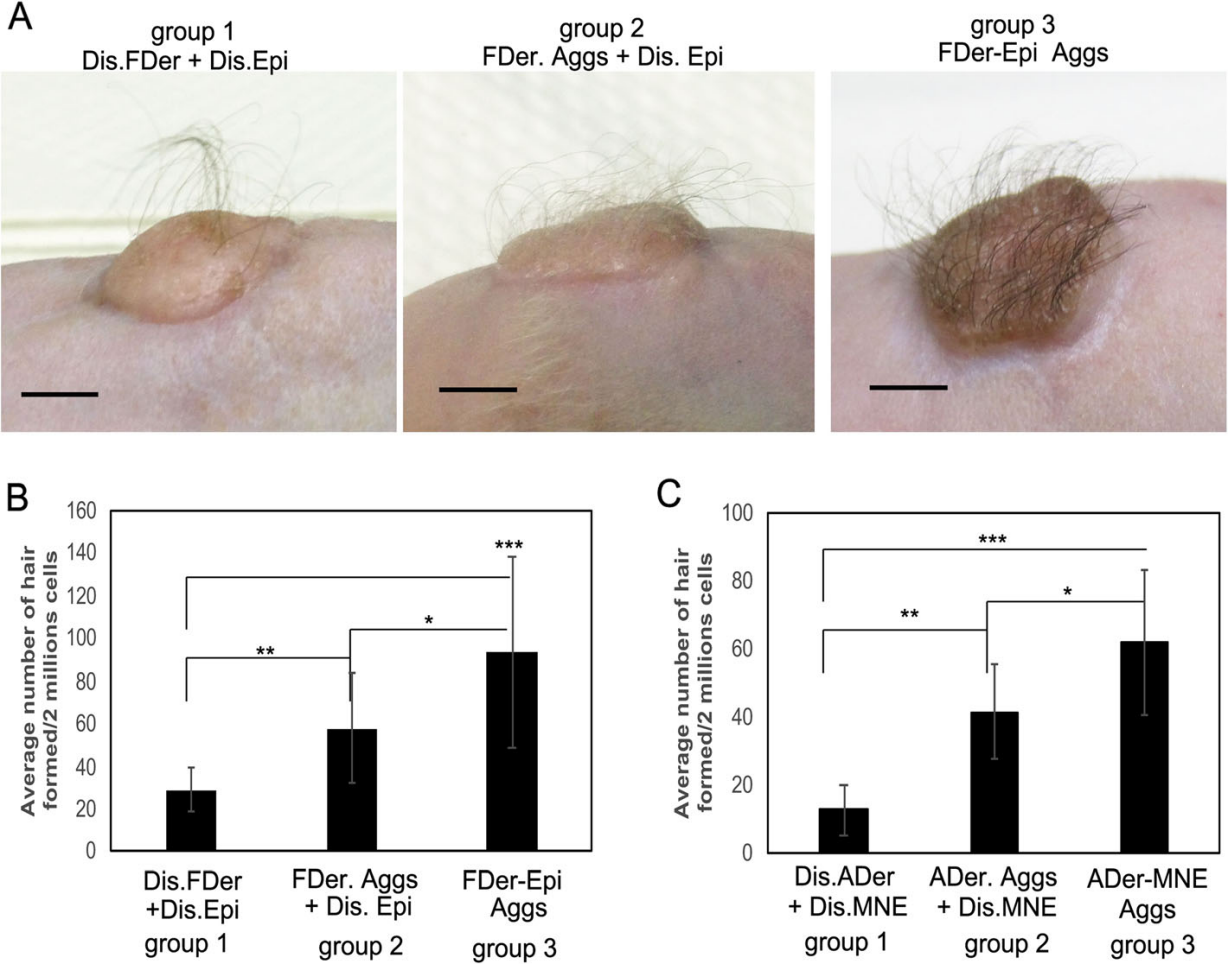
Aggregated dermal and epidermal cells produce more hair follicles in transplants. **a** Representative images of grafts at 3 months after transplantation of three groups of cells (group 1: Dis.FDer + Dis.Epi, group 2: FDer. Aggs + Dis.Epi, group 3: FDer-Epi Aggs), detailed information about each group is described in condition 1 of Table 1. **b** Quantification of the average number of hair follicles formed in each graft in **a**; a total of 4 mice (*n* = 4) were counted in each group. **c** Quantification of the average number of hybrid hair follicles formed in each patch assay, performed with subcutaneous injection of three groups of adult dermal cells (ADer) mixed with MNE cells (group 1: Dis.ADer + Dis.MNE, group 2, ADer.Aggs + Dis.MNE, group 3: ADer-MNE Aggs); detailed information about each group is described in condition 3 of Table 1, a total of 4 mice (*n* = 4) were counted in each group. *P < 0.05,**P < 0.01;***P < 0.005 when two groups were compared as indicated. Please note the different scale used on the *y*-axis of **c**

Hair follicles are composed of epidermal (epithelial) and dermal (mesenchymal) compartments, and their crosstalk plays an important role in their morphogenesis [12, 22]. We hypothesized that pre-aggregating dermal and epidermal cells in vitro could enhance hair follicle formation in vivo. To test that hypothesis, we initially used dermal cells derived from fetal scalp tissue, which had previously been shown to have strong hair regeneration potential [17], combined with foreskin-derived epidermal cells (condition 1 in Table 1). Three groups of those same populations of cells (Table 1) were grafted onto the dorsal skin of nude/nude mice. Representative images of mice from those three groups 3 months after the grafting are shown in Fig. 1a. Hair shaft formation was observed in all three groups, which was confirmed by histological analysis (Additional file 1: Figure S3). We observed that more hairs formed in groups 2 (FDer. Aggs) and 3 (FDer-Epi Aggs) with the greatest number appearing in group 3 (*P* < 0.005), and the dissociated cells without aggregation of group 1 formed the lowest number of hairs (Fig. 1a, b). This result suggests that the pre-aggregation of dermal and epidermal cells in vitro enhances hair regeneration in vivo. To further confirm this result, we used dermal cells derived from adult scalp tissues (condition 2, Table 1). In that case, the efficiency of hair formation was too low to detect any difference among the three groups, although high numbers of hairs formed dermal-epidermal aggregates (group 3, Additional file 1: Figure S4). To evaluate the grafting potential of murine cells analogous to human foreskin-derived epidermal cells, we used MNE cells combined with adult dermal cells (condition 3, Table 1). The three groups of cells from condition 3 (Table 1) were assessed by patch assays. The results mirrored what was observed in Fig. 1b, i.e., the pre-aggregation of adult dermal cells with MNE cells (ADer-MNE Aggs) produced the highest number of hairs (Fig. 1c). Taken together, these results suggest that suspension cultures of dermal and epidermal cells initiate a crosstalk between them, which boosts the hair regeneration potential in vivo.

### Dermal-epidermal aggregation enhances the WNT pathway

**Fig. 2:**
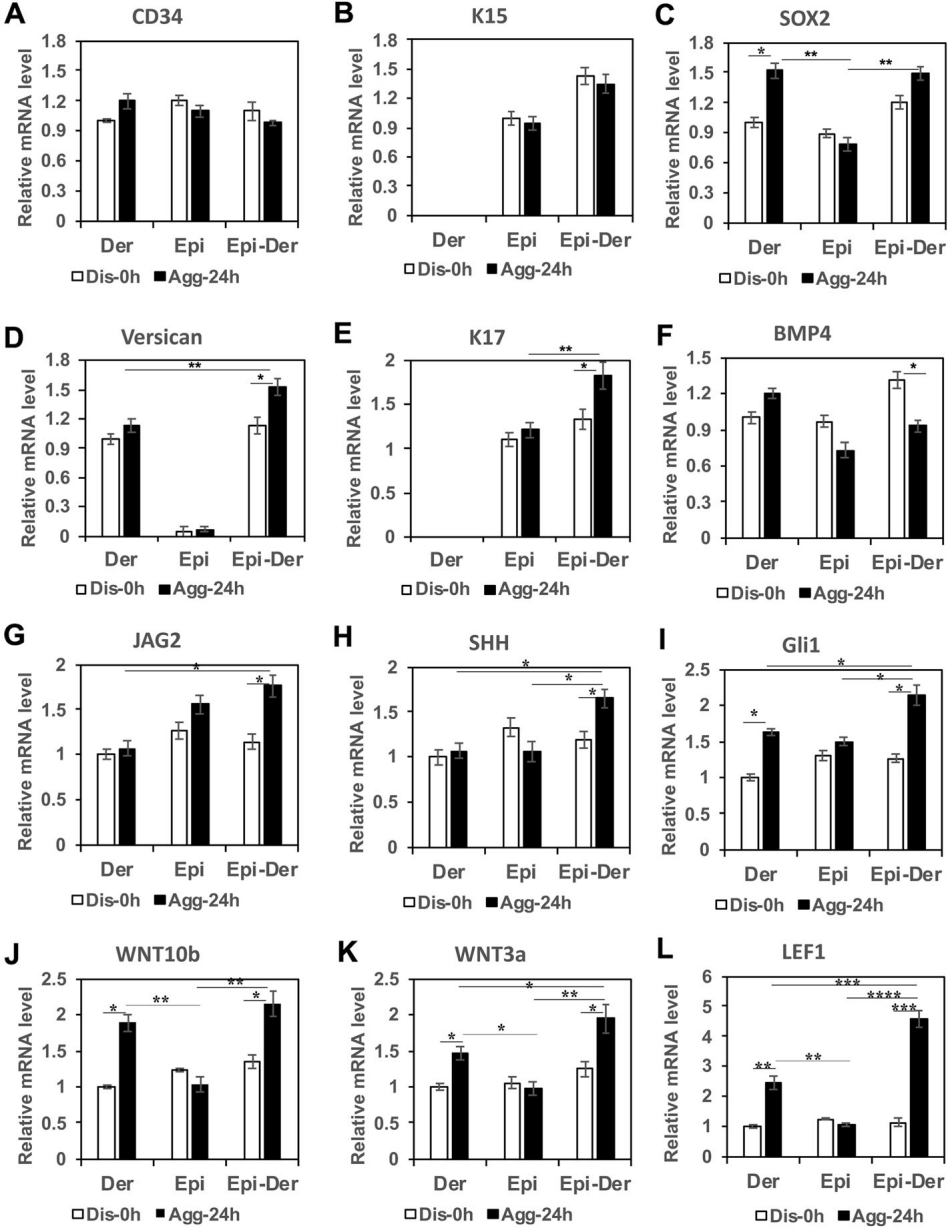
Aggregation of dermal and epidermal cells enhances the expression of WNT pathway-related genes. **a–l** Dissociated dermal (Der), epidermal (Epi), or mixed dermal-epidermal (Epi-Der) cells at a 1:1 ratio were collected for total mRNA extraction (Dis-0 h) without aggregation or were placed into suspension for 24 h to form aggregates, after which the aggregates were harvested at 24 h (Agg-24 h) for total mRNA extraction. A total 2 × 10^5^ cells were used for this experiment. Total mRNAs from all conditions were analyzed for the expression of genes as indicated by RT-PCR, and the relative expression level of each gene was normalized with the housekeeping gene 36B4. The experiment was repeated three times (*n* = 3). Significance between groups labeled with black lines was established by *P* values as indicated: *P < 0.05,**P < 0.01;***P < 0.005, ****P < 0.001

To understand the molecular basis for the observed enhanced hair regeneration following the aggregation of dermal and epidermal cells, we tested the expression of genes known to be involved in hair follicle development and of markers in dermal, epidermal, and dermal-epidermal aggregates formed in suspension culture for 24 h (Agg-24 h) compared with the corresponding dissociated cells (Dis-0 h) without aggregation. First, we analyzed the expression of two hair follicle stem cell markers, CD34 and Keratin 15 (K15) [41, 42]. There was no clear difference in CD34 expression among these cells with different conditions (Fig. 2a). K15 was not expressed in the dermal cells and was not induced in epidermal or dermal-epidermal aggregations (Fig. 2b). Next, two dermal papilla cell markers, SOX2 and versican [43, 44], were analyzed. The expression of SOX2 was induced by dermal aggregation (*P* < 0.05) but not by epidermal aggregation or dermal-epidermal aggregation (Fig. 2c). The expression of versican, which was not expressed in epidermal cells, was induced in dermal-epidermal aggregations (Fig. 2d) (*P* < 0.05). The expression of Keratin 17 (K17), a specific hair lineage gene that is normally expressed in the outer root sheath of hair follicles [45], was induced in dermal-epidermal aggregations (Fig. 2e). Finally, the expression of genes encoding major signaling molecules, including BMP, Notch, Sonic hedgehog, and WNT pathway factors, which are involved in regulating hair morphogenesis and development [26], was studied. As shown in Fig. 2f, expression of the BMP4 gene was downregulated in dermal-epidermal aggregates, while the expression of Notch (JAG2), Sonic hedgehog (SHH, GLi1), and WNT pathway factors (WNT10b, WNT3a, and LEF1) was significantly increased in dermal-epidermal aggregates compared to dermal-dermal or epidermal-epidermal aggregates (Fig. 2g–l). Especially, the levels of expression of these WNT-related genes, WNT3a, WNT10b, and LEF1, were increased dramatically (*P* < 0.005) in the dermal-epidermal aggregates, suggesting that dermal-epidermal cell crosstalk impacts mainly the WNT pathway. Taking these data together indicates that the epidermal-dermal aggregation induces the expression of hair follicle signature genes and activates signaling pathways, especially the WNT pathway, which plays crucial roles in hair development.

### Dermal-epidermal cells interact to form two major types of aggregates in vitro

**Fig. 3:**
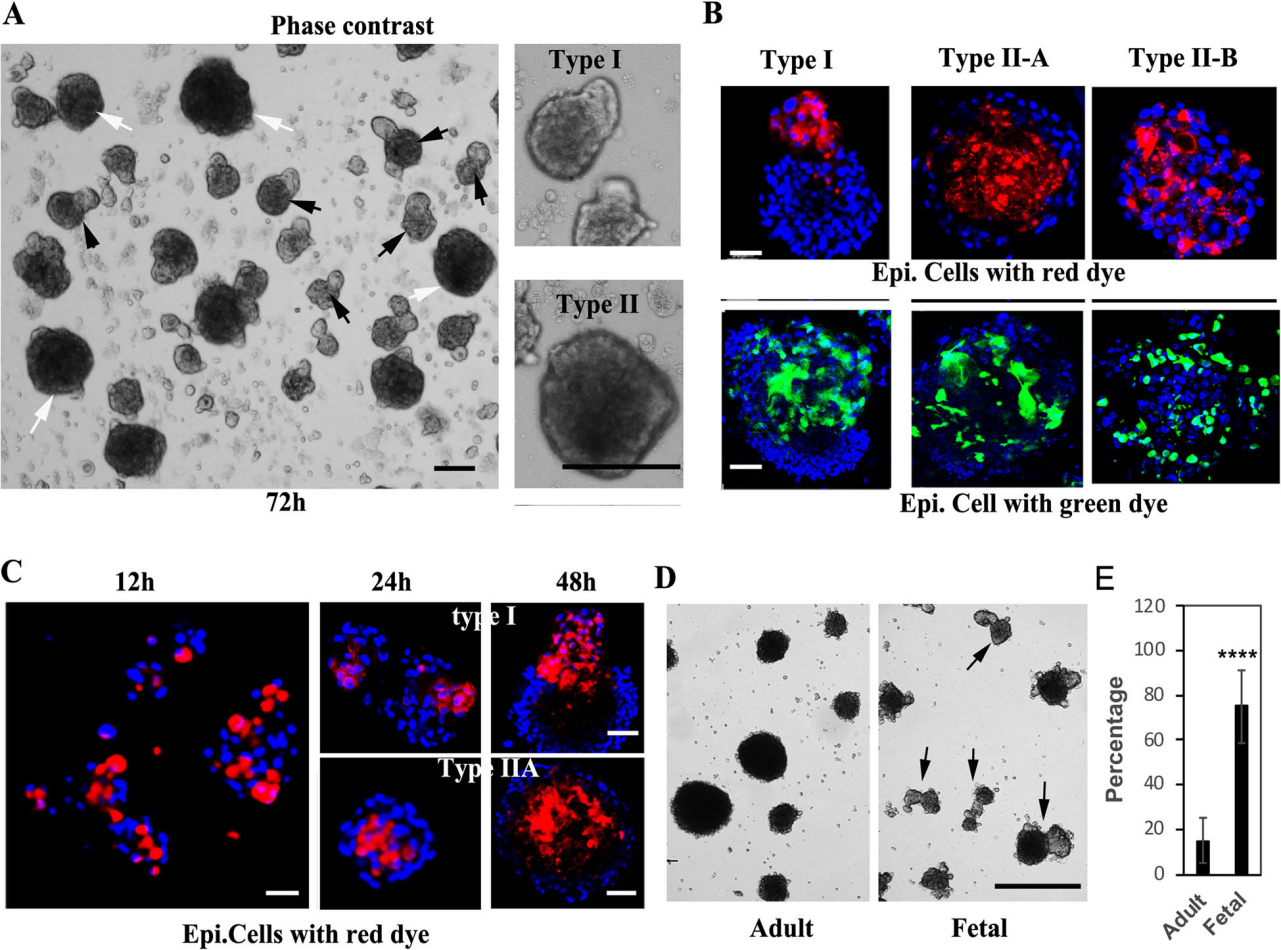
Fetal scalp-derived dermal cells and foreskin-derived epidermal cells form aggregates with a polarized pear-shape structure. **a** Representative images of aggregates formed from a 3-day (72 h) culture of fetal scalp-derived dermal cells mixed with newborn foreskin-derived epidermal cells at a 1:1 ratio in suspension. White arrows indicate type II aggregates with a spherical morphology and black arrows indicate type I aggregates with a pear-like shape. Higher magnification images of type I and type II aggregates are shown in the right panels. Bars = 200 μm. **b** Representative images of 3-day aggregates formed from mixtures of fetal scalp-derived dermal cells and membrane dye labeled foreskin-derived epidermal cells. The upper panels show aggregates with red dye labeled epidermal cells and the lower panels show aggregates with green dye labeled epidermal cells. Nuclei are stained with DAPI (blue). Bars = 50 μm. **c** Representative images of aggregates formed from fetal scalp-derived dermal cells mixed with red dye labeled foreskin-derived epidermal cells, collected at 12, 24, and 48 h after suspension culture. Nuclei are stained with DAPI (blue). Bars = 50 μm. **d** Representative images of 2d (48 h) aggregates formed using adult scalp-derived dermal cells (Adult) or fetal scalp-derived dermal cells (Fetal) with the same foreskin-derived epidermal cells. Black arrows indicate type I aggregates. Bar = 500 μm. **e** Quantification of the percentage of type I aggregates formed in **d**; ****P < 0.001 when the fetal group is compared with the adult group

We observed that dermal cells alone form one type of sphere in 3D suspension cultures (Additional file 1: Figure S5). However, when starting with a mixture of epidermal and dermal cells, two major morphological types of aggregates formed: Type I aggregates are pear-shaped (white arrows, Fig. 3a) and Type II aggregates are spherically shaped (black arrows, Fig. 3a), similar to dermal cell spheres (Additional file 1: Figure S5). In order to understand how cells organize to form these two distinct types of morphologies, we mixed labeled foreskin-derived epidermal cells (with their membranes dyed red or green) with unlabeled fetal-derived dermal cells and then examined the aggregates that formed after 3 days of suspension culture. We found that the protrusion portion of type I aggregates consisted of either red or green labeled cells (Fig. 3b), indicating that the stalk structure is formed by the epidermal cells. The type II aggregates were further distinguished into two subtypes: for type IIA, the red or green labeled epidermal cells were present in the inner sphere, and for type IIB, the labeled epidermal cells were randomly distributed in the sphere (Fig. 3b).

Next, we characterized the time course of formation of type I and type II aggregates as monitored by fluorescence confocal microscopy at 12, 24, and 48 h. We found that the two types of morphology developed as early as 24 h after seeding (Fig. 3c). To test whether the aggregate morphology indicates scalp dermal cell regenerative potential, we assayed the same epidermal cell populations with either fetal- or adult scalp-derived dermal progenitor cells. As shown in Fig. 3d, significantly greater numbers of type I aggregates were formed in the fetal cell group, indicating that the formation of type I aggregates may correspond to trichogenic potential since there is a much higher efficiency of hair regeneration by fetal cells in vivo (Fig. 1a, b and Additional file 1: Figure S4A-B).

### Type I aggregates express hair follicle markers

**Fig. 4:**
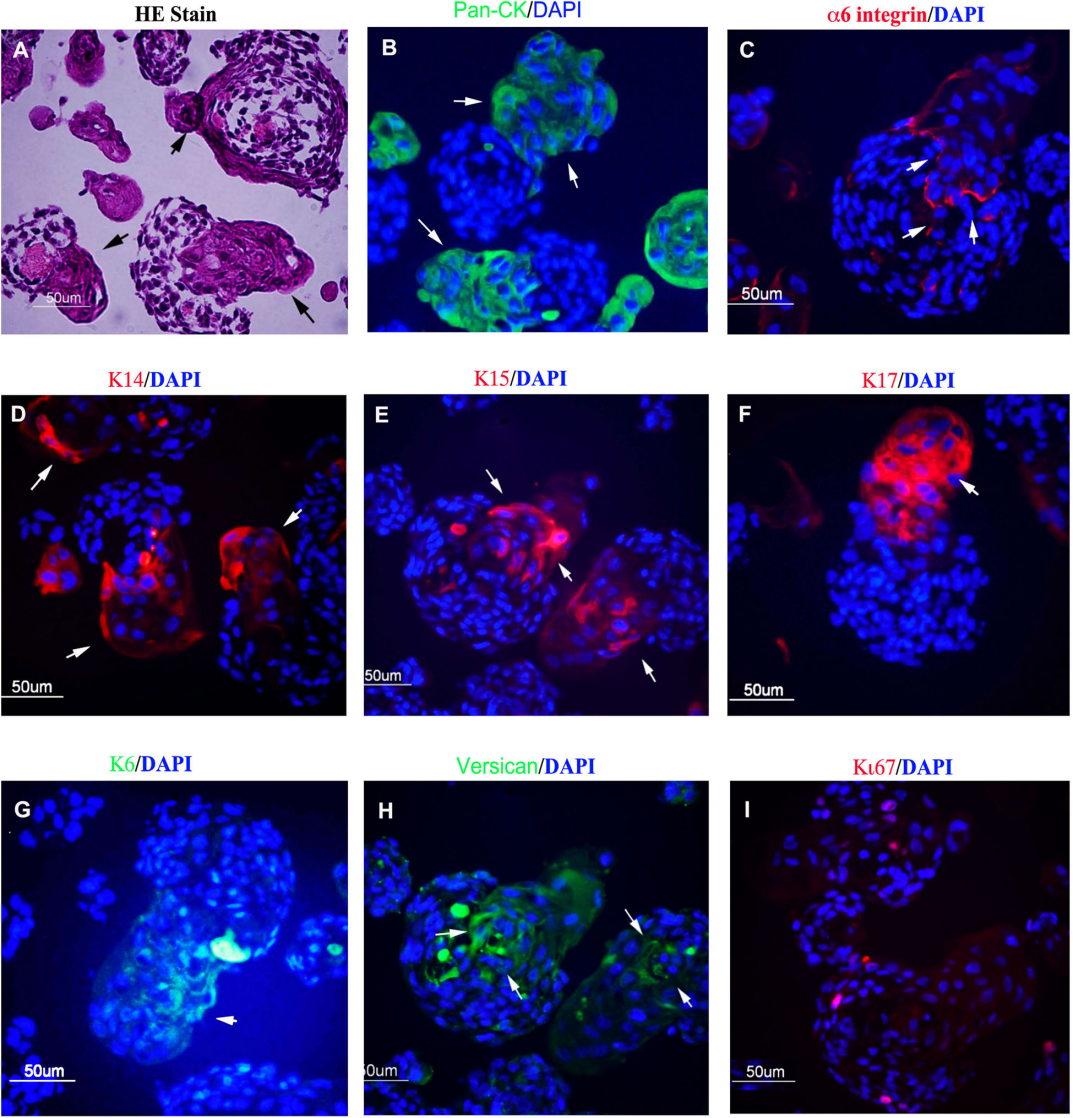
Type I aggregates present hair peg-like structures. **a–i** Three-day dermal-epidermal cell aggregates from Fig. 1a were harvested and processed for H&E staining for histological analysis and for IF staining (red or green) for analysis of the expression of proteins as indicated. Nuclei are stained with DAPI (blue); black arrows indicate epithelized structures; white arrows indicate positive staining of the corresponding proteins. Bars = 50 μm

Since the formation of type I aggregates reflects the regeneration potential of scalp dermal cells and the morphology or organization of epidermal and dermal cells of type I aggregates is reminiscent of the structure of early-stage hair follicles [13], we wondered if type I aggregates represent nascent hair follicles. Therefore, we characterized the structure of type I aggregates formed at 3 days by performing histological analysis and IF staining. H&E staining of type I aggregates showed keratinization of the protrusion structures (black arrows, Fig. 4a), which were stained positively for the human pan-CK antibody (green, white arrows, Fig. 4b). The epidermal stalks expressed keratin 14 (K14) (white arrows, Fig. 4d), a marker of the basal layer of the epidermis while the lower part of the epidermal protrusions expressed keratin 15 (K15) (white arrows, Fig. 4e), a hair follicle bulge stem cell marker. Type I aggregates also expressed the hair follicle markers keratin 17 (K17) (white arrow, Fig. 4f) and keratin 6 (K6) (white arrow, Fig. 4g) in the epithelium protrusions. The basement membrane demarking the epidermal and dermal components of the protrusions expressed a6 integrin (white arrows, Fig. 4c). Staining for versican, a mesenchymal cell marker expressed in the dermal papilla, was found in the region right under the protrusions (arrows, Fig. 4h), which suggested that the type I aggregates might contain dermal papilla-like structures. Finally, we found that staining for the proliferation marker, Ki67 (red dots, Fig. 4i), appeared in dermal cells of aggregates at 3 days, suggesting that cells in the aggregates were actively growing. Taken together, these data suggest that type I aggregates resemble hair pegs, an early stage of hair follicles when the epidermal cells grow down into the dermis after the phase of dermal condensation [22].1

### WNT pathway activation is essential to hair peg-like formation during the dermal-epidermal aggregation process

**Fig. 5:**
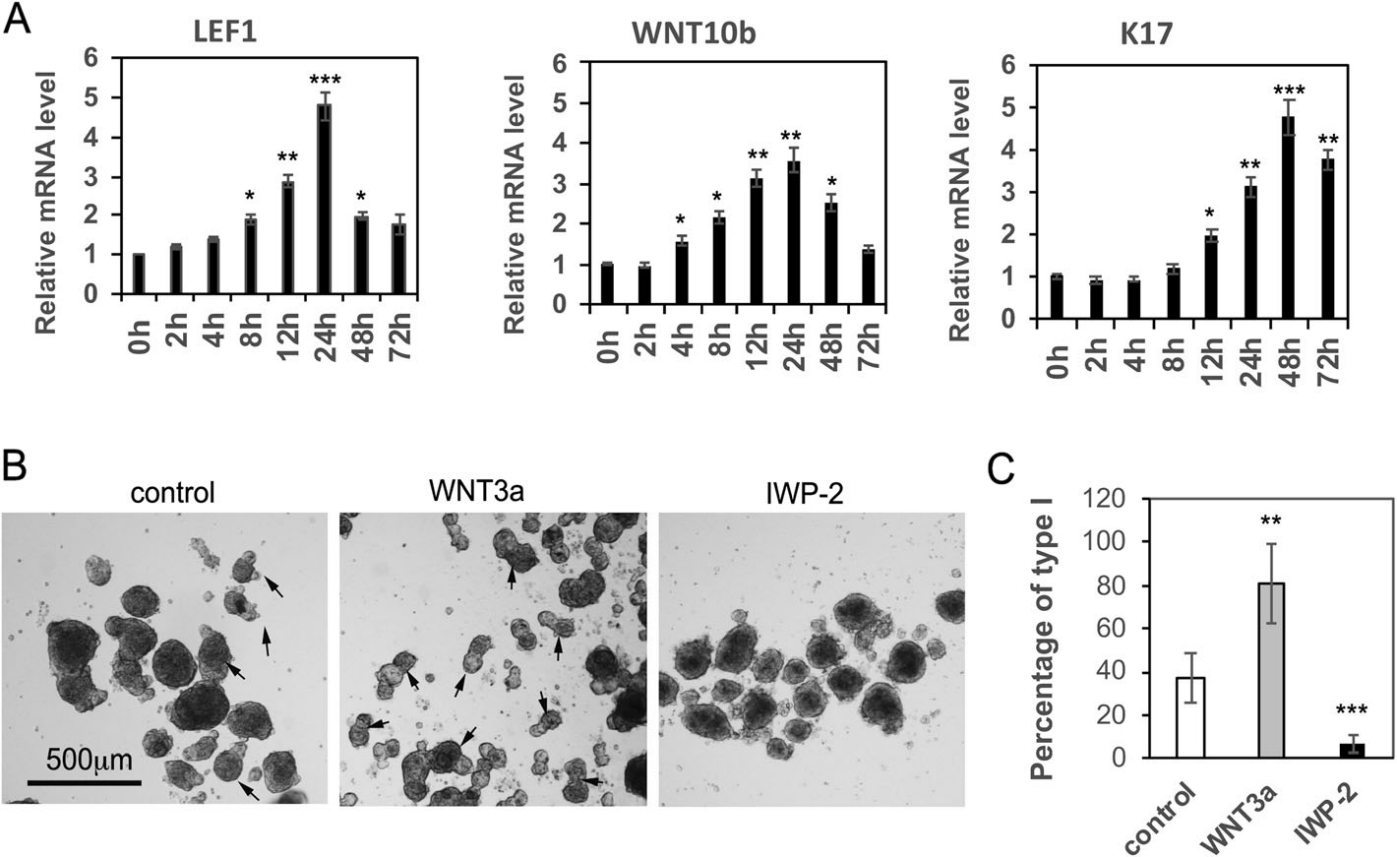
Activation of the WNT pathway is essential for the formation of type I aggregates. **a** Fetal scalp-derived dermal cells mixed with foreskin-derived epidermal cells at a 1:1 ratio were cultured in suspension and harvested at different time points as indicated for RT-PCR analysis of LEF1, WNT10b, and K17 expression. *P < 0.05, **P < 0.01, and ***P < 0.005 when compared with the group at 0 h. **b** Representative images of 48 h aggregates formed from fetal scalp-derived dermal cells mixed with foreskin-derived epidermal cells at a 1:1 ratio in suspension cultures in the presence of DMSO (control), WNT3a or IWP-2. Bar = 500 μm. Black arrows indicate type I aggregates. **c** Quantification of the percentage of type I aggregates formed in **b**, **P < 0.01, ***P < 0.005 when compared to the control group

As shown in Fig. 2, the WNT pathway is significantly upregulated during the dermal and epidermal aggregation process. Next, we determined when the WNT pathway was activated during the aggregation process. Aggregates were formed from fetal scalp dermal progenitor cells and foreskin epidermal stem cells in suspension cultures and were collected at different time points for analysis of gene expression patterns. RT-PCR analysis of the expression of LEF1, WNT10b, and the hair follicle marker K17 is shown in Fig. 5a. A significant elevation of WNT pathway factors starts from 4 to 6 h after suspension and LEF1 expression reaches its highest point at 24 h, while the expression of K17 reached a maximum at 48 h after suspension (*P* < 0.005). To further test the importance of the WNT pathway in the formation of type I aggregates, we added WNT3a recombinant protein to the culture medium. We found that the addition of WNT3a increased the percentage of type I aggregation formation (*P* < 0.01, Fig. 5b,c). Conversely, adding the WNT inhibitor IWP-2 blocked the formation of type I aggregates (*P* < 0.005, Fig. 5b,c). These data underscore the importance of the WNT pathway to the formation of hair peg-like structure in vitro.

## Discussion

Hair follicles consist of epidermal (epithelial) and dermal (mesenchymal) compartments, and thus epidermal-mesenchymal cell interactions play an essential role in regulating their morphogenesis and growth [22, 25, 44]. Because effective crosstalk between those two compartments has also been considered to be a key for successful laboratory reconstitution of hair follicles [46], we hypothesized that the pre-aggregation of scalp dermal and epidermal cells in vitro might potentiate hair follicle regeneration in vivo*.* Indeed, in the present study, we show that the transplantation of dermal-epidermal aggregates which had been formed previously in vitro resulted in a greater number of hair follicles compared to the transplantation of mixtures of dissociated dermal and epidermal cells or dermal aggregates mixed with dissociated epidermal cells (Fig. 1). These results suggest that the pre-aggregation of dermal-epidermal cells probably initiates crucial crosstalk between dermal and epidermal cells, which results in the enhancement of hair follicle formation after transplantation in vivo. Indeed, we found that dermal-epidermal aggregation promotes the expression of the hair follicle lineage gene K17 and the dermal papilla signature gene versican. Importantly, we found that the aggregation of epidermal and dermal cells downregulates BMP signaling and activates NOTCH, SHH, and WNT pathway-related factors, which are the main signals that control epithelial and mesenchymal cell interactions during hair morphogenesis [27, 47]. Notably, the dermal-epidermal aggregation dramatically increased the expression of WNT ligands WNT3a and WNT10b and the WNT downstream target LEF1 (Fig. 2), and the expression of LEF1 increased nearly fivefold in epidermal-dermal aggregates compared to dissociated cells. Considering that WNT signaling is the dominant pathway in hair development and differentiation [22, 27], we conclude that the strong activation of WNT signaling elicited by the pre-aggregation of dermal-epidermal cells promotes hair formation after transplantation in vivo.

We observed that epidermal-dermal cell suspensions lead to the formation of two kinds of aggregates, type I and type II, that have distinct morphologies (Fig. 3). Type I aggregates are pear-shaped, resembling the hair peg, an early stage of hair follicle during morphogenesis [22]. From cell-tracing studies, we found that the type I aggregates are well-organized structures with distinct epidermal cells and dermal cell compartments where epidermal cells form the stalk structure (Fig. 3b, c). Further characterization of type I aggregates revealed their histological and IF staining features that are characteristic of hair follicles (Fig. 4). Such findings are similar to those of hair follicle organoids as regenerated by droplet cultures [16, 17]. Illustrating the importance of the Wnt pathway, we show here that the addition of WNT3a protein to the growth medium enhanced the formation of type I aggregates, and conversely, treatment with IWP2 to inhibit WNT processing and secretion [48], blocked their formation (Fig. 5b, c). Therefore, we conclude that the mixture of scalp dermal cells and epidermal cells in suspension can activate the WNT pathway, which leads to the development of type I aggregates, a nascent hair follicle organoid, in vitro. Future work will attempt to generate a single hair follicle in vivo by transplanting a single type I aggregate.

Recently, it was shown that the overexpression of LEF1 significantly enhanced human hair follicle formation in a 3D-printed mold [49]. A significant induction of LEF1 expression was observed in epidermal-dermal aggregates in this study. The formation of air-peg-like structures in type I aggregates (as early as 24 h) (Fig. 3) is concomitant with the peak of LEF1 expression (Fig. 5). Moreover, we could efficiently and reproducibly reconstitute hair follicles in vivo after transplanting fetal scalp dermal and foreskin epidermal aggregates for 24 h (Fig. 1). Taking these results together suggests that LEF1 could serve as a biomarker for hair regeneration, and a significant induction of LEF1 expression in epidermal-dermal aggregates likely was a major contributor to the formation of hair follicle organoids.

In agreement with previous studies, the present study also clearly showed that it is still a big challenge to efficiently regenerate hair follicles both in vivo and in vitro using dermal cells derived from adult scalp tissue. Due to ethical considerations, the use of cells derived from fetal tissues is not recommended, especially for clinical applications. Therefore, future work will be directed at developing trichogenic cells from induced pluripotent stem cells (iPSCs) and to reprogram adult cells into fetal-like cells to replace the fetal cells in our system.

## Conclusion

In summary, our system is able to form a large number of hair follicle organoids within 24 h using a simple suspension of fetal scalp dermal progenitor cells and adult foreskin epidermal stem cells. The present study provides a system for many applications such as testing the trichogenic potential of hair stem cells and for performing large-scale screening for compounds that enhance hair regeneration, as well as for studying general phenomena involved in mesenchymal-epithelial interactions.

## Supplementary information

**Additional file 1: Figure S1.** Foreskin-derived epidermal cells at passage 3 are able to form holoclones. **Figure S2.** Flowchart of the suspension assay. **Figure S3.** More hair follicles are found in reconstituted skin formed from the transplantation of dermal-epidermal cell aggregates. **Figure S4.** The efficiency of hair regeneration by adult scalp-derived dermal cells combined with foreskin-derived epidermal cells is low. **Figure S5.** Dermal cells alone formed spheres in suspension cultures. **Table S1.** Oligo sequences for RT-qPCR analysis.

## Data Availability

The dataset used and/or analyzed during the current study are available from the corresponding author upon reasonable request.

## Abbreviations

BSA: Bovine serum albumin
FBS: Fetal bovine serum
H&E: Hematoxylin and eosin
IF: Immunofluorescence
iPSC: Induced pluripotent stem cells
IWP2: Inhibitor of WNT Production-2
K17: Keratin 17
LEF1: Lymphoid enhancer binding factor 1
MNE: Mouse newborn epidermal
O.C.T: Optimal cutting temperature
PBS: Phosphate-buffered saline
PFA: Paraformaldehyde
