# Supplementary material for: Pre-aggregation of scalp progenitor dermal and epidermal stem cells activates the WNT pathway and promotes hair follicle formation in in vitro and in vivo systems
Source: Stem Cell Res Ther. 2019 Dec 19;10:403. doi: 10.1186/s13287-019-1504-6 (PMC6921573; doi:10.1186/s13287-019-1504-6)
Supplement: Supplementary file 1 — Additional file 1: Figure S1. Foreskin-derived epidermal cells at passage 3 are able to form holoclones. Figure S2. Flowchart of the suspension assay. Figure S3. More hair follicles are found in reconstituted skin formed from the transplantation of dermal-epidermal cell aggregates. Figure S4. The efficiency of hair regeneration by adult scalp-derived dermal cells combined with foreskin-derived epidermal cells is low. Figure S5. Dermal cells alone formed spheres in suspension cultures. Table S1. Oligo sequences for RT-qPCR analysis. [file 13287_2019_1504_MOESM1_ESM.docx]

**Supplemental Figures and Figure Legends**

**
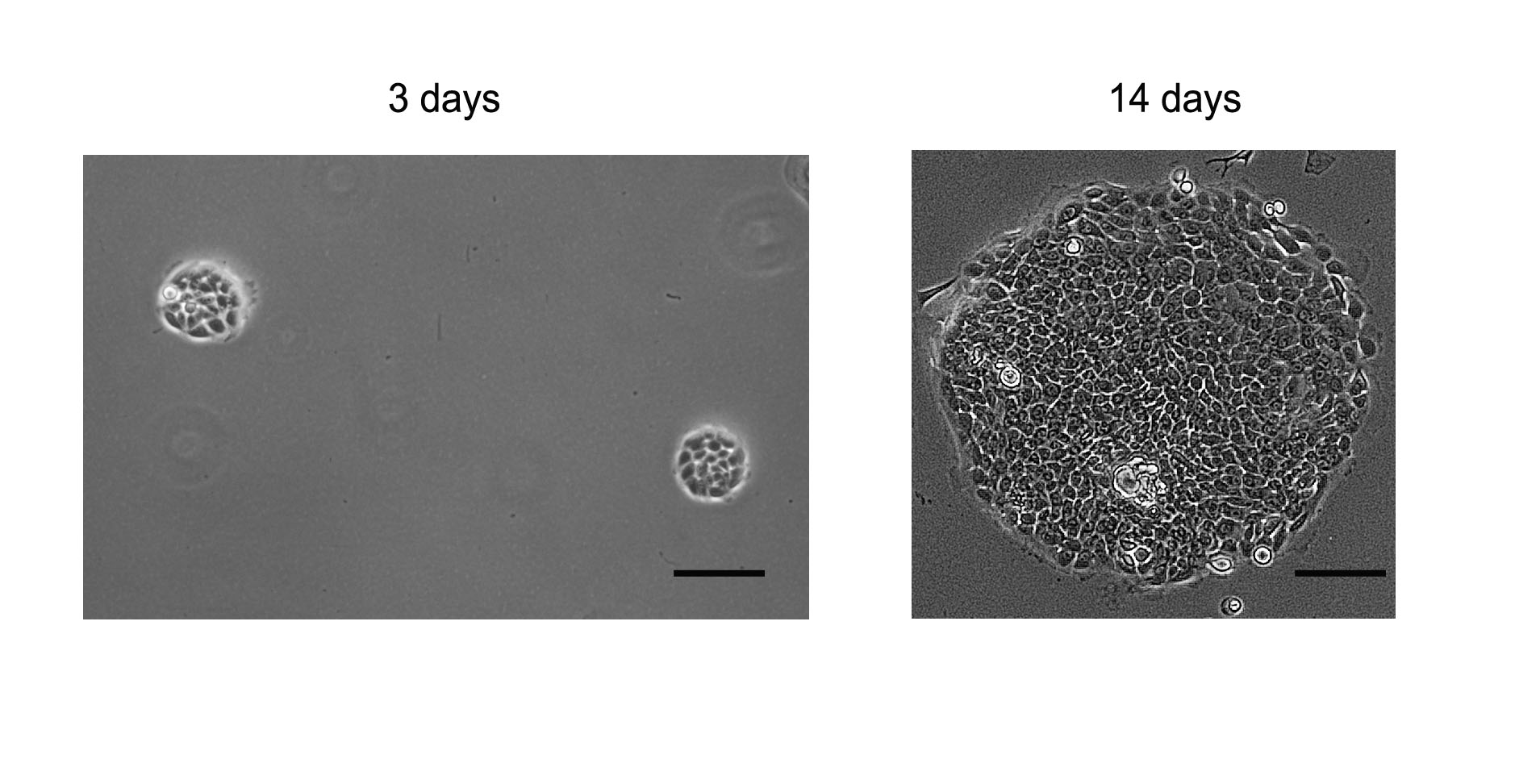
**

**Figure S1.** Foreskin-derived epidermal cells at passage 3 are able to form holoclones.

500 dissociated foreskin-derived epidermal cells at passage 3 were seeded into 100 mm culture dishes, and representative images of the colonies formed are shown at 3 days (left panel) and at 14 days (right panel) after seeding. Bars = 100 μm.


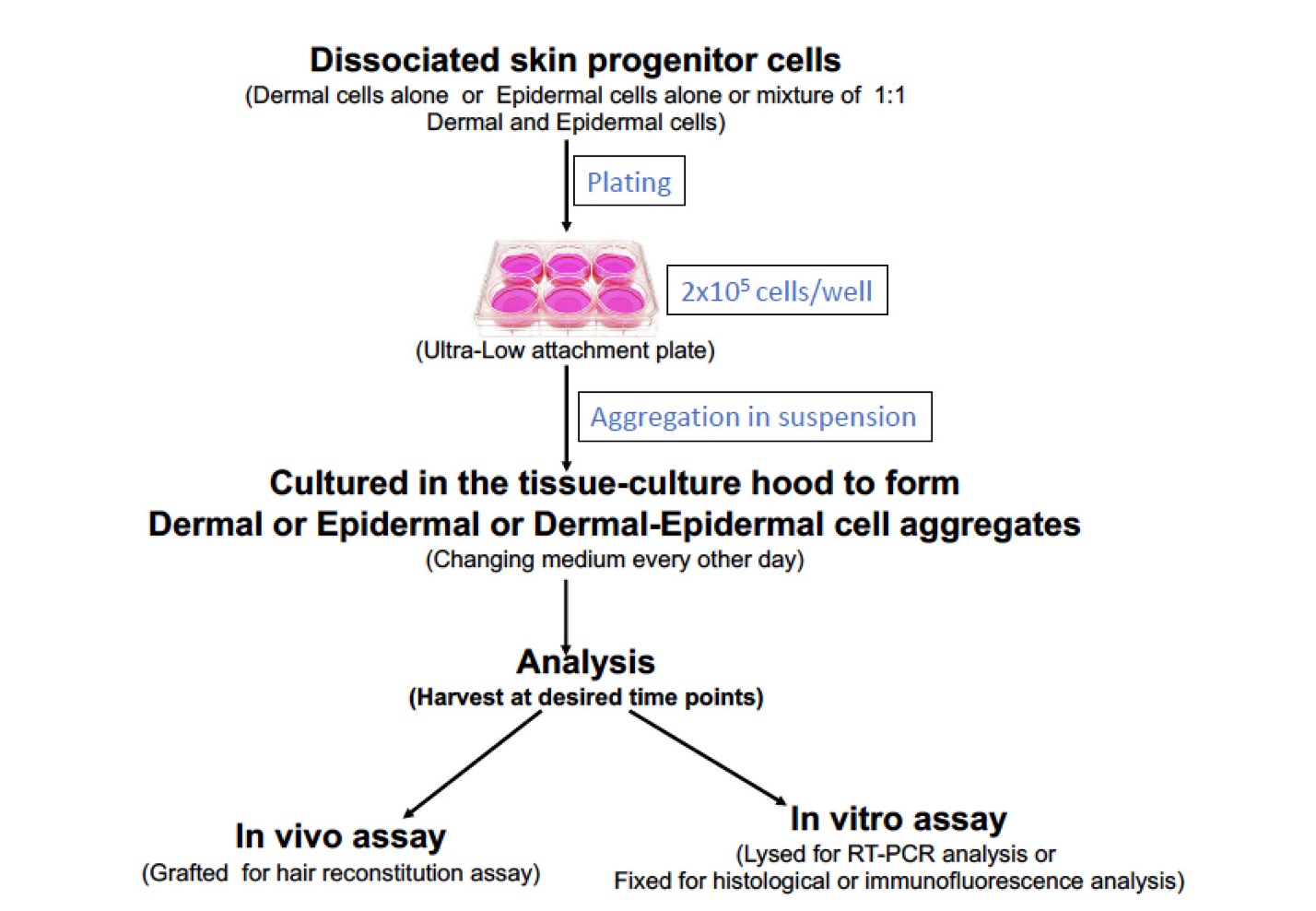


**Figure S2.** Flowchart of the suspension assay

**
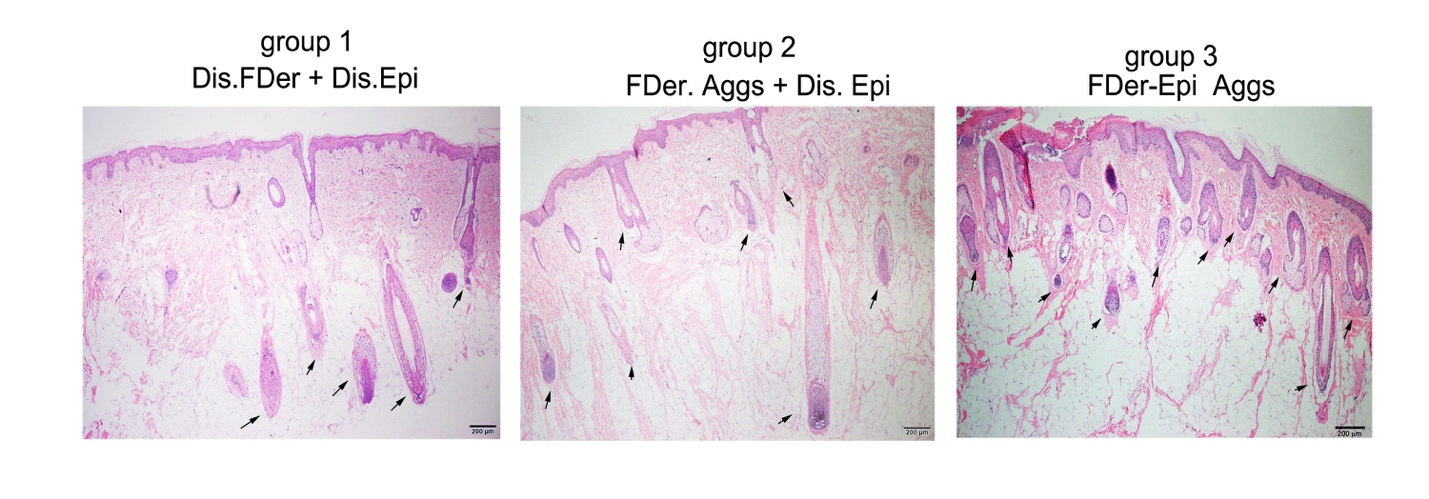
**

**Figure S3.** More hair follicles are found in reconstituted skin formed from the transplantation of dermal-epidermal cell aggregates.

Histological analysis (H&E stain) of reconstituted skins formed from the transplantation of three different groups of cells shown in Fig. 1A. Black arrows indicate hair follicles. Bars = 200 µm.


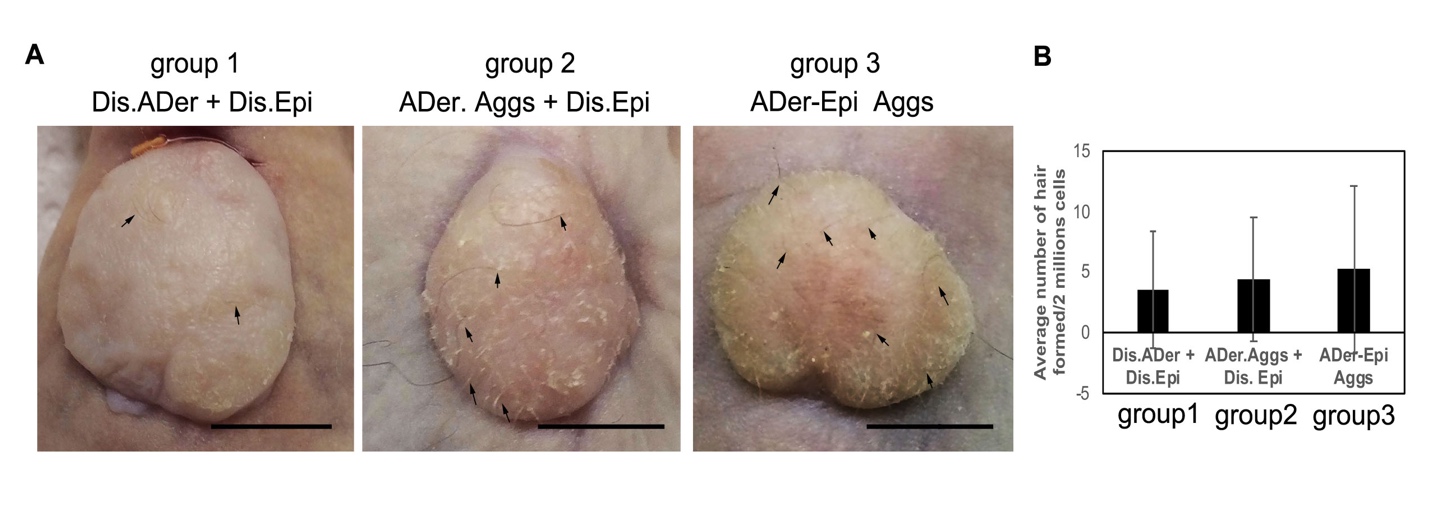


**Figure S4.** The efficiency of hair regeneration by adult scalp-derived dermal cells combined with foreskin-derived epidermal cells is low.

**A.** Representative images of grafts at 3 months after transplantation of three groups of Adult dermal cells (ADer) combined with foreskin-derived epidermal cells (Epi) (group 1: Dis.ADer + Epi, group 2: ADer. Aggs + Dis.Epi, group 3: ADer-Epi Aggs), detailed information about each group is described in condition 2 of Table 1. **B**. Quantification of the average number of hair follicles formed in each graft in **A**, a total of 4 mice (n=4) were counted in each group. Bars = 5 mm.

**
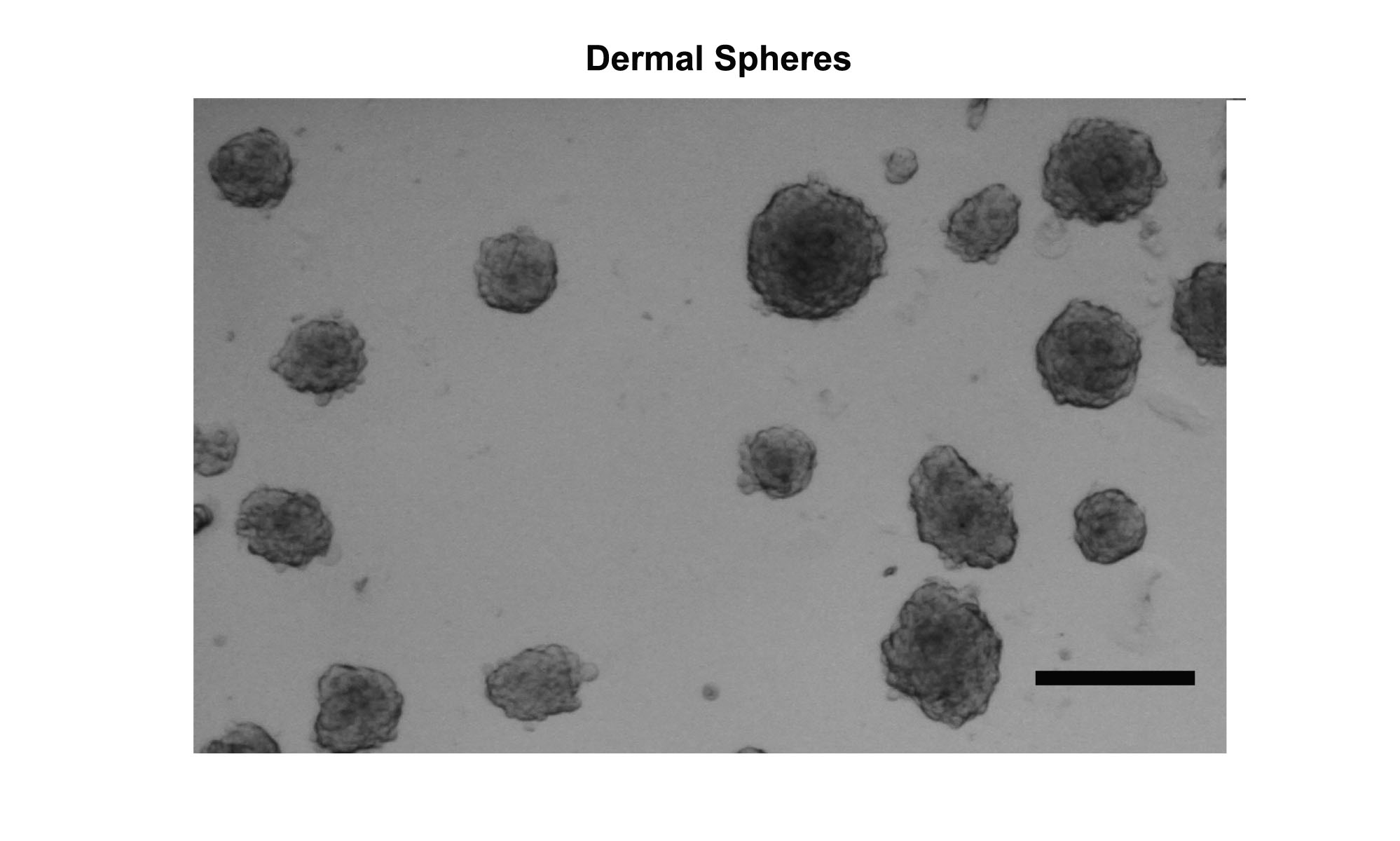
**

**Figure S5.** Dermal cells alone formed spheres in suspension cultures.

2x10^5^ dissociated fetal scalp-derived dermal progenitor cells at passage 3 were plated in each well of ultra-low attachment 6-well plates, and representative images of spheres were taken as shown above. Bar = 200 μm.

**Table S1. Oligo sequences for RT-qPCR analysis**

| **Gene Name** | **Forward** | **Reverse** |
| --- | --- | --- |
| CD34 | TTTACCGTTTCTTCCCTGTA | AAGTCCACAGTGTCTTGGAG |
| SOX2 | CCACCTACAGCATGTCCTAC | GAGTGGGAGGAAGAGGTAAC |
| Versican | AGAAGACACCATCCACACTC | TGTCTCTCTTCCCAGTCATC |
| Wnt3a | GTGACTTCCTCAAGGACAAG | GGCACCTTGAAGTAGGTGTA |
| Wnt10b | CATCCAGGCACGAATGCGA | CGGTTGTGGGTATCAATGAAGA |
| Lef-1 | AGAACACCCCGATGACGGA | GGCATCATTATGTACCCGGAAT |
| K17 | GGTGGGTGGTGAGATCAATGT | CGGCATCCTTGCGGTTCTT |
| K15 | AAACTTCTTCCTCCACCTTT | CCTAGCAGAAGAAGCTGAGA |
| BMP4 | AGCCATGCTAGTTTGATACC | TAAAGATCCCGCATGTAGTC |
| JAG2 | AATGAGTGTGAAGGGAAGC | CAGTCGTTGACGTTGATATG |
| SHH | AGAGGAGTCTCTGCACTACG | GTAGTACACCCAGTCGAAGC |
| GLI1 | CCAAACGCTATACAGATCCT | CCACTGTAGAAATGGATGGT |
